# Lymph Node Cellular Dynamics in Cancer and HIV: What Can We Learn for the Follicular CD4 (Tfh) Cells?

**DOI:** 10.3389/fimmu.2018.02233

**Published:** 2018-09-27

**Authors:** Antigoni Poultsidi, Yiannis Dimopoulos, Ting-Fang He, Triantafyllos Chavakis, Emmanouil Saloustros, Peter P. Lee, Constantinos Petrovas

**Affiliations:** ^1^Department of Surgery, Medical School, University of Thessaly, Larissa, Greece; ^2^Tissue Analysis Core, Immunology Laboratory, Vaccine Research Center, NIAID, NIH, Bethesda, MD, United States; ^3^Department of Immuno-Oncology, Beckman Research Institute, City of Hope Comprehensive Cancer Center, Duarte, CA, United States; ^4^Institute of Clinical Chemistry and Laboratory Medicine, Technische Universität Dresden, Dresden, Germany; ^5^Department of Internal Medicine, Medical School, University of Thessaly, Larissa, Greece

**Keywords:** cancer, HIV, Tfh cells, lymph nodes, follicles

## Abstract

Lymph nodes (LNs) are central in the generation of adaptive immune responses. Follicular helper CD4 T (Tfh) cells, a highly differentiated CD4 population, provide critical help for the development of antigen-specific B cell responses within the germinal center. Throughout the past decade, numerous studies have revealed the important role of Tfh cells in Human Immunodeficiency Virus (HIV) pathogenesis as well as in the development of neutralizing antibodies post-infection and post-vaccination. It has also been established that tumors influence various immune cell subsets not only in their proximity, but also in draining lymph nodes. The role of local or tumor associated lymph node Tfh cells in disease progression is emerging. Comparative studies of Tfh cells in chronic infections and cancer could therefore provide novel information with regards to their differentiation plasticity and to the mechanisms regulating their development.

## Introduction

Given the important role the lymphatic system has in combating foreign pathogens, changes in Lymph Node (LN) architecture/cellularity have been recognized in a variety of infectious diseases. In Human Immunodeficiency Virus (HIV) infection, LNs play a central role for disease pathogenesis. Early studies revealed the covert infection of CD4 T cells in the LN and its role in the depletion of these cells throughout disease progress (1, 2). From a prognostic point of view, the degree of follicular structure damage has been used for classification of disease progress (3, 4). Current vaccine strategies targeting the humoral arm of the immune system have revealed the need for a comprehensive understanding of follicular dynamics. Given the role of T follicular helper (Tfh) cells as an HIV reservoir (5) and as critical “helpers” in the development of antibodies (6), understanding their biology is of great interest.

Besides infectious diseases, LNs are involved in various forms of neoplasia, either as metastasis or as primary disease sites (i.e., lymphomas). It is of critical importance to distinguish between infectious etiologies of lymphadenopathy vs. neoplastic causes (7). In non-hematopoietic neoplastic disease, LNs are involved in disease progression (a) as part of the regional disease, contributing to local morbid phenomena when infiltrated by the tumor, (b) as metastatic disease *per se*–(N status in the Tumor Node Metastasis staging system)–affecting the treatment/management of patients, and (c) as a mediator for propagating further distant metastasis. Although LN involvement has major prognostic implications for the patient and is thus incorporated in the staging strategies of neoplasms (8), the clinical management of the lymphatic system draining a malignancy is an area of ongoing research and trends are shifting accordingly. For example, in breast cancer patients attention has been drawn to the Sentinel Lymph Node (SLN) (9), which is defined as the first LN or group of LNs that interstitial fluid and cells from the tumor microenvironment pass through on their route to the venous circulation via lymphatic vessels (10–12). Despite the different etiology and specific pathways/molecular factors operating selectively in cancer or infectious diseases, the comparative analysis of LN immune-dynamics could provide important information regarding the development and maintenance of Tfh cells.

## Lymph nodes: organization, Tfh cells

LNs provide the site of initiation of adaptive immune responses and are strategically placed along lymphatic vessels (13). Antigen presenting cells (APCs) initiate immune responses via interactions with T and B cells that gain access to specific regions of the LN (14). Functionally, the LNs can be separated into lobules (13). The structural backbone of the lobule is comprised of Fibroblastic Reticular Cells (FRCs) and their fibers, which support the parenchyma, provide routes for the migration of lymphocytes, and facilitate the interaction between lymphocytes and APCs (4). The inter-follicular regions of the cortex and the paracortex are mainly populated by T cells, which gain access to the parenchyma by migrating through high endothelial venules (HEVs), following the CCR7/CCL19, CCL21 axis (4). These cells interact with dendritic cells (DCs) that have reached the LN via the afferent lymphatic vessels and HEVs (4). Primary follicles (located in the cortex) contain mainly naïve B cells, whereas secondary follicles are recognized by the formation of a germinal center (GC) (13). GCs, the antibody production factory of the body, are populated by antigen stimulated B cells, follicular dendritic cells (FDCs), Tfh cells, and macrophages- among other cell types (15, 16). FDCs can present antigens and stimulatory signals to GC B and T cells, as well as produce CXCL-13, the ligand for CXCR5 (17), while tingible body macrophages are capable of phagocytizing dying cells (13).

Tfh cells provide critical signals for the activation, isotype switching, affinity maturation, and differentiation of B cells into memory B cells and plasma cells via surface bound receptors (i.e., PD-1, ICOS, CD40) (18, 19) and secreted factors like IL-21 and IL-4 (20, 21), that support the GC responses by regulating the differntiation of both Tfh and B cells through the activation of STAT signaling pathways (21–25). The spatial organization of Tfhs cells is regulated, at least, by (i) chemokine gradients (i.e., CXCL-13 and CXCL-10/IP-10, a chemokine produced by macrophages and acting on CXCR3) enabling their trafficking toward GC (17, 26). and (ii) function of signaling pathways mediating their retention within the follicular/GC areas. GC homing is accomplished via downregulation of CCR7 and upregulation of CXCR5- a process mediated by Bcl-6, a critical transcription factor for Tfh cell differentiation (27, 28). Once inside the GC, S1PR1 family receptors aid in Tfh cell retention in GCs- this is accomplished by downregulation of S1PR1 and upregulation of S1PR2 (29–31). Dynamic positioning inside the GC is influenced by the local production of factors, one of which is CXCL-12/SDF-1 that acts on CXCR4 (32, 33). The unique PD-1^hi^CXCR5^hi^ phenotype has been widely used for the identification of Tfh cells (34, 35). In line with this, imaging studies have shown the highly skewed localization of PD-1^hi^ CD4 T cells within the GC areas (GC-Tfh cells) (35–37). High expression per cell (judged by Mean Fluoresense Intensity-MFI) of other surface receptors (like ICOS and TIGIT), is selectively found on the vast majority of GC-Tfh cells (35, 37, 38). The differential expression of surface receptors like CD150 and CD57 can further delineate GC-Tfh subpopulations (35, 37, 39). For example, Tfh cells expressing lower levels of CD150 (SLAM) secrete higher levels of IL-4 and are thought to be more differentiated than Tfh cells expressing higher levels of CD150 (37, 39). The presence of PD-1^dim^CXCR5^hi^ (non-GC) Tfh (34), the differential expression of CXCR3 (Th1-like Tfh cells) (40, 41), or Tfh master regulators like Bcl-6 (5, 28, 35), further adds to the heterogeneity of the Tfh pool. We should emphasize, though, that different follicular CD4 T cell subsets are presumably exposed to different local signals within the follicle. Delineation of these signals, as well as the connection between phenotype and function of Tfh cell subsets, is an important step toward the comprehensive understanding of Tfh cell biology and their role in human diseases.

A separate group of CD4 T cells located in the follicle/GC- and particularly the T-B area border (42)- are T follicular regulatory (Tfr) cells (43), which possibly originate from thymic T regulatory (Treg) cells, after adaption of their gene expression profile to include -apart from FoxP3- factors and receptors expressed in Tfh cells, such as Bcl-6 and CXCR5 (38, 44, 45). A mutual regulation between Tfh and GC B cells through the function of receptor/ligand axes (such as CD40/CD40L, ICOS-ICOSL) has been proposed (46–48). In a similar manner, Tfr cells can suppress GC reactivity (43) either by altering such mutual regulation- a process mediated in part by CTLA-4 (49)- or by directly affecting B or Tfh cells (43, 50). Although they represent a small minority of follicular CD4 T cells, the presence of Tfr cells aids in limiting the GC response to prevent uncontrolled B cell proliferation and the consequences thereof, such as the production of antibodies that recognize “self” antigens (37, 44).

## Lymph nodes in neoplasms

### The concept of SLNs

From an immunological perspective, SLNs are the site where tumor antigen loaded APCs encounter naïve T and B cells, leading to the generation of immune responses against neoplasms (51–53). A relatively smaller distance from the primary tumor site presumably increases the possibility for the SLN to be affected by the tumor than downstream draining LNs (DLNs) are, potentially leading to the variable immune responses observed in DLNs depending on the distance from the primary tumor (54). Factors which modulate these responses may tip the balance from control to tolerance/spread of the neoplasm (51–53). Most research has focused on SLNs in the context of melanoma and breast cancer patients, but knowledge is expanding about SLNs in other types of neoplasms -such as genitourinary, pulmonary, and gastrointestinal tumors (11). However, accurate identification of SLNs can be very challenging (55). Rerouting of lymphatic flow or the presence of tumor cells in the subcapsular sinus may affect the ability to detect the SLN with the help of dyes (55, 56). Lymphangiogenesis and alterations of lymph flow dynamics induced by the tumor may also alter lymphatic drainage (55). Therefore, a false negative SLN (up to 9.8% in breast cancer) could result in under-staging and mistreating patients (57, 58).

### Structural alterations of SLNs induced by tumors

**Figure 1:**
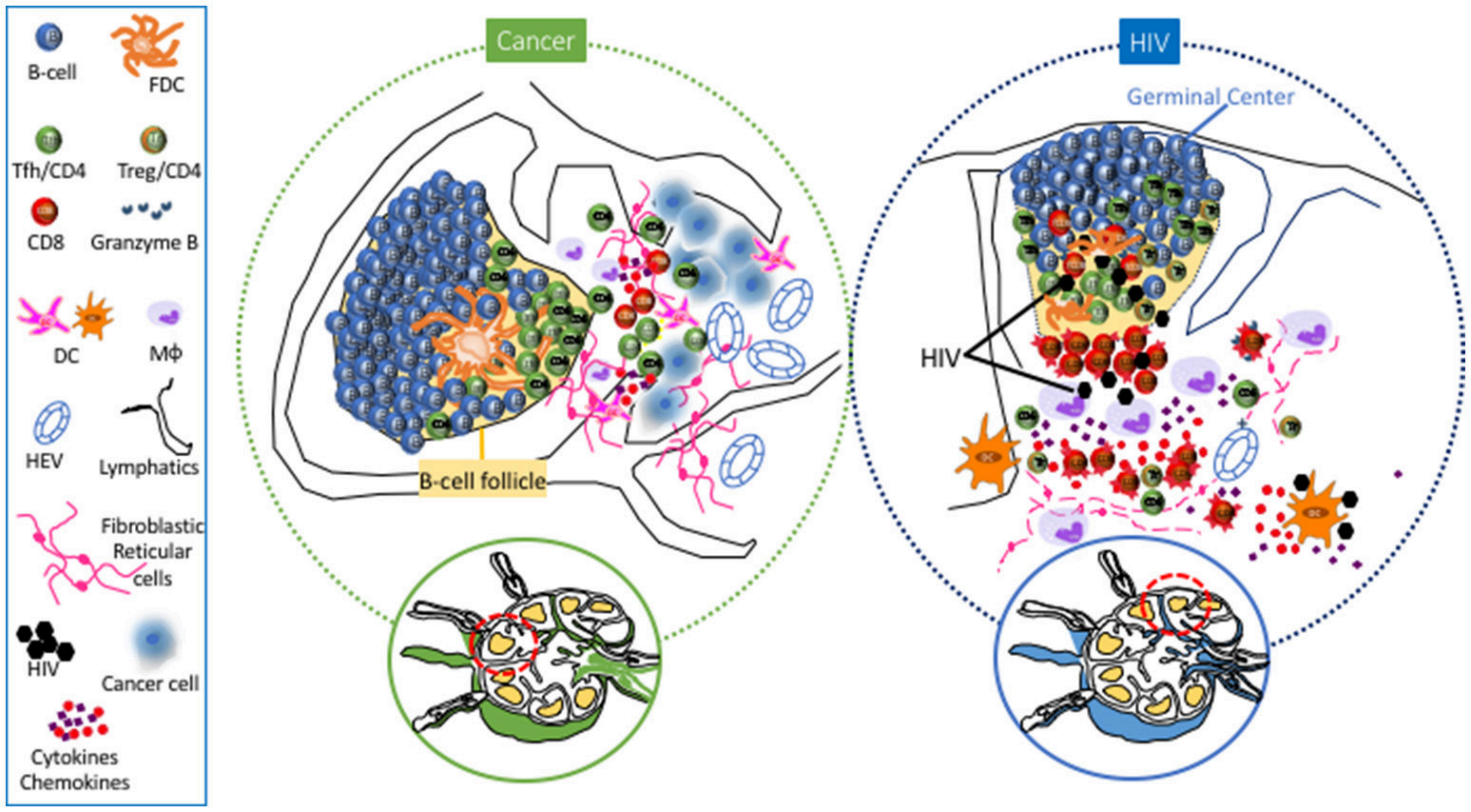
Main Lymph Node cell dynamics in cancer (left) and HIV (right). Cancer causes lymphangiogenesis and vasculature reorganization in SLNs. Metastasis is associated with accumulation of DCs and rercuitment of moncytes/macrophages and NK cells. Follicular hyperplasia associated with the development of Tfh cells has also been observed in SLNs and particularly in TLSs. Chronic HIV/SIV infection is characterized by extensive loss of FRC and naive CD4 T cells, accumulation of monocytes/macrophages, particularly in the area surrounding the follicle, as well as accumulaiton of effector CD8 T cells in the extrafollicular area (T cell zone). Within the follicle, accumulation of Tfh cells is associated with loss of their polarized positioning in the light zone, loss of FDC network and accumulation of follicular Treg CD4 T cells (Tfr).

Tumors can affect structural components in the SLNs and DLNs even before metastasis to these sites has occurred, creating an environment fostering tumor cell invasion to the SLN (59–61). Major structural SLN changes have been described, related to (a) increased lymphatic drainage from the tumor to the LNs (55), which can induce biophysical remodeling/changes of the LN matrix (62) and potentially lead to the activation of signaling pathways [i.e., transforming growth factor (TGF)-β pathway] associated with tumor spreading/induction of immune suppression in the LNs, in a similar fashion to what has been observed in primary tumor sites (63), (b) increased lymphangiogenesis and angiogenesis induced by vascular endothelial growth factors (VEGFs) originating from the tumor environment, which can ultimately contribute to the spreading of tumor cells to the SLN and beyond (55, 60, 64–66), and (c) a “flatter” morphology of the endothelial cells of the HEVs, which can potentially lead to impaired access of naïve T cells to the LN parenchyma (53). Overall, these structural changes in the lymphatics and the vasculature can set the stage for future metastasis of cancer cells to SLNs (Figure 1).

### Non-follicular immune dynamics in SLNs

Apart from structural changes, modulations of immune cell subsets have also been observed to precede actual metastasis of tumor cells to SLNs. Several studies suggest a compromised capacity to induce a “favorable” Th-1 response against the tumor due to (i) decreased DC density and clustering in paracortical LN regions (67–70) and (ii) compromised DC function (60, 70–72). Conversely, other studies have advocated increased presence of activated DCs in SLNs, prior to the appearance of metastatic cells (73–75). However, transition to a mature DC phenotype and Th-1 cytokine response was noted after metastasis of breast tumor cells to the SLN, possibly reflecting antigenic stimulation against these cells (72) (Figure 1). Emerging studies have investigated the role of LN NK cells (76) and monocytes/macrophages in anti-tumor immunity (77, 78) and their targeting for adjuvant immunotherapies that could improve treatment of patients with metastatic cancer. Regarding adaptive immunity, alterations in cell types with prognostic implications (68) have been observed in SLNs and DLNs. Reduced numbers of CD4 and CD8 T cells (60, 64, 68) with an immunosuppressed profile (79) was found in SLNs, a profile associated with accumulation of FoxP3+ Treg CD4 cells in LNs harboring metastases (80–83) and worse prognosis/more widespread nodal disease in melanoma, breast, and gastric cancer (82–85). Various cytokines (GM-CSF, IL-2) are being investigated as a way to reverse this immune suppression and assist in the immune system's effort to combat the neoplastic cells (53).

### Follicular dynamics in SLNs and tertiary lymphoid structures (TLSs)

In contrast to extrafollicular cell dynamics, much less is known about follicular/B cell dynamics in the context of neoplastic disease. In SLNs, B cells- via the secretion of VEGF-A- could induce lymphangiogenesis and angiogenesis (61, 86, 87), potentially promoting the spread of tumors via lymphatics (55). However, an extended lymphatic network can lead to increased recruitment of DCs from the periphery to the LN (86), which could ultimately benefit the development of anti-tumor adaptive immunity. A trend toward improved 5-year survival was noted in melanoma patients, whose SLNs demonstrated follicular hyperplasia/GC accumulation (88). Besides their prognostic value for disease-free survival in breast cancer patients (89), SLN B cells is the source of affinity matured B cell clones that produced anti-tumor immunoglobulins detected in the blood (90). Investigation of such antibodies could potentially lead to the recognition of tumor antigens recognized by the immune system, which can subsequently be targeted in the context of immunotherapies (90). Furthermore, these findings imply that development of tumor-specific Tfh cells could be a critical factor for an effector response to a tumor. The progressive differentiation of Tfh cells within the follicular area, associated with differential localization and an orchestrated production of IL-21 and IL-4, provide critical signals for the isotype switching and differentiation of GC B cells by modulating transcription factors like Bcl-6 and Blimp-1 (21, 24, 91). In a mouse tumor model, accumulation of Tfh cells was noted in DLNs, along with a concomitant increase in IL-4 produced by these cells (92). On the other hand, recent studies have shown a beneficial role of IL-21 in cancer immunotherapy strategies (93, 94), possibly by modulating CD8 T cell response (95–97). Therefore, Tfh cells could potentially support antitumor immunity in ways extending past the help they provide to B cells.

The role of B cell infiltration in primary tumors is not clear (98, 99), with studies showing both a negative (100, 101) or a positive (102–105) effect on the antitumor immune responses. However, B cells contribute to the formation of tertiary lymphoid structures (TLSs) - defined as accumulations of lymphocytes in proximity to the primary tumor—which are associated with better prognosis (99) (Figure 1). Similar to B cells, an increased presence of Tfh cells in the primary tumor site has been associated with better clinical outcomes in breast (106) and non-small cell lung carcinoma (107). High expression of molecules like CXCL-13 and IL-21 (106, 108) by TLS associated Tfh cells contributes to the formation/organization of TLSs in the primary breast tumor and potentially contribute to the immune systems' reaction (109). Presumably, TLSs facilitate the *in situ* production and secretion of anti-tumor antibodies that could represent a mechanism to maximize the efficiency of adaptive immunity against tumors (99). A variety of regulatory cell subsets have the ability to influence Tfh cell function. In breast cancer, LN Treg cells can promote malignancy through a TGFβ-1 mediated upregulation of the oncogenic receptor IL17rb (110). A coevolution of Treg cells and CXCL-13^hi^ Tfh cells in the TLSs was found, with the ratio between these two populations being a critical factor for tumor control by benefiting the development of an anti-tumor humoral response (109). Furthermore, the presence of myeloid-derived suppressor cells within the LN could potentially be a negative regulator for Tfh cells (111, 112), adding to the complexity of the regulation of these cells. Characterization of relevant cytokine producers and their spatial positioning within anatomically separated LN areas would be highly informative in understanding their potential role in regulating Tfh cell dynamics in SLN and TLSs.

Several reports have been focused on the characterization of circulating CXCR5^hi^ CD4 T (cTfh) cell subsets as a counterpart of the LN bona fide Tfh cells (113, 114). The lineage origin of cTfh cells and their direct association to LN Tfh cells is not clear (115, 116). Lower cTfh cells in the blood of hepatocellular carcinoma patients were associated with worse prognosis (117), while a higher frequency of “Th-1” CXCR3^hi^ cTfh cells was negatively associated with survival in gastric cancer (118). In breast cancer, a higher frequency of “exhausted” Tim-3^hi^ cTfh cells associated with higher expression of PD-1 per cell base was found -interestingly, *in vitro* blocking of Tim-3 increased the production of IL-21 and CXCL-13 by peripheral blood mononuclear cells (119). Future investigation of cTfhs in cancers of different etiology could provide important information regarding their use as a biomarker, as well as their relationship to LN or TLS Tfh cells.

## Follicular immune dynamics: lessons from HIV/SIV (simian immunodeficiency virus)

### Structural alterations

HIV infection leads to dramatic and progressive changes of LN architecture, especially evident during the chronic phase of infection (4). In reality, the degree of tissue damage has been used for the staging of disease (120). A major contributor to this damage is the extensive deposition of collagen (fibrosis) in the extrafollicular area (121), a process facilitated by increased levels of secreted TGF-β1 from accumulated Treg cells (122, 123) and the activation of spatially associated fibroblasts (124, 125). Fibrosis leads to a vicious circle of naïve T cell pool and FRC network depletion (126, 127)- a network that provides the scaffold for cell migration (128) and vital signals for the recruitment (CCR7) (129, 130) and survival (IL-7) (130, 131) of naïve T cells (Figure 1). LN damage is associated with the persistent immune activation and tissue inflammation found in HIV/SIV (4). Despite the partial normalization of immunological parameters- such as CD4 counts, immune activation, and suppressed viremia- LN structure abnormalities persist in combination antiretroviral therapy (cART)-treated individuals (132–134), presumably affecting the development and function of LN relevant T cells -such as Tfh cells- in the context of new infections or vaccination (36).

### Non-follicular immune dynamics

Besides tissue architecture, HIV/SIV infection has a major impact on the cellular dynamics within the extrafollicular areas. Monocytes/macrophages that express low levels of CD4 and other HIV coreceptors (135) can contribute to HIV/SIV pathogenesis by (i) supporting the viral reservoir, particularly in advanced disease or immunocompromised states (136, 137), and (ii) secreting inflammatory mediators like IL-6 and IL-10 (138), which play an important role in the development of GC responses (139). The accumulation of monocytic-lineage and plasmacytoid dendritic cells (pDCs) in LNs during acute SIV infection (140–143) is followed by their impaired function (leading to decreased production of cytokines like IFN-a and IL-12, which *in vitro* support T cell proliferation) during the chronic phase of infection (144–146). Despite the loss of both pDCs and myeloid DCs (mDCs) from lymphoid tissues and blood in chronic infection, LN-derived mDCs retain their functionality, especially the induction of Treg cells- an important regulator of Tfh cell function and GC reactivity (147, 148). Chronic HIV/SIV is characterized by the relative loss of LN CD4 cells- mainly attributed to loss of naïve CD4 T cells (39, 126, 149)- accompanied by an increased frequency of effector CD8 T cells (149) (Figure 1). Besides the direct killing of infected CD4 T cells, the cellular and molecular mechanisms regulating the LN T cell dynamics in HIV/SIV are not well understood. Structure damage, immune activation, inflammatory signals, and altered tissue chemokine gradients could all play an important role in this process. Recent studies have shown that chronic HIV/SIV infection is associated with sequestration of monocytes/macrophages around the follicular areas (150). Their possible role in LN CD8 T cell dynamics is supported by their (i) correlation with LN CD8 T cell in chronic SIV (149), (ii) spatial proximity to accumulated CD8 T cells in LN areas (149, 150), and (iii) potential to change local chemokine gradients through the secretion of chemokines like CXCL-9 and CXCL-10 (IP10) (151, 152), ligands for the CXCR3 receptor that is broadly expressed on LN CD8 T cells (149). Such altered chemokine gradients could contribute to LN T cell dynamics by modulating their (i) recruitment from the circulation (153, 154) and (ii) intra lymph node trafficking (26).

### Follicular dynamics

Understanding the follicular/GC- and particularly Tfh cell- immune dynamics in HIV/Simian Immunodeficiency Virus (SIV) infection is of great importance for (i) the identification of molecules/pathways associated with the development of broadly neutralizing antibodies that could inform the design of novel vaccine strategies targeting relevant GC cell populations and (ii) understanding the establishment and maintenance of a major viral reservoir (5), even in cART treated donors (155). To this end, the non-human primate (NHP) SIV model has provide invaluable information regarding the Tfh cell dynamics during infection. A relatively delayed development of Tfh cells during acute SIV has been described in peripheral LNs (39). Interestingly, different kinetics between spleen and LN associated Tfh cells has been found, indicating a differential regulation of Tfh cells in different lymphoid organs (156, 157). Chronic HIV/SIV infection is associated with an altered (a) frequency (39), (b) function and signaling (39, 156), (c) molecular profile (39, 158), and (d) localization/distribution within the follicular areas (159) of Tfh cells (Figure 1). The dynamics of LN Tfh cells- associated with follicular hyperplasia in the LNs and hypergammaglobulinemia in the plasma (39, 160–162)- have been linked to progression to AIDS (162, 163), as well as to immune activation and associated cytokines—such as IL-6 and IFN-γ (19, 39, 161). The dependence of Tfh cells on immune activation and tissue inflammation is further supported by their downregulation in cART individuals (5, 160) and by the lack of accumulation of Tfh cells in LNs from infected African Green Monkeys (AGMs) (149), a natural host of the virus with no signs of immune activation (164). Apart from the altered frequency, SIV infection has a significant impact on the molecular signature of Tfh cells- characterized by upregulation of IFN-γ and TGF-β related genes (39)- indicating an increased response of Tfh cells to relevant stimuli and a role of TGF-β as regulator of Tfh cell dysfunction in chronic infection. The combination of (i) an increased expression of CXCL-13 in Tfh cells (39), (ii) a favorable phosphorylation of STAT3 (a positive regulator of Tfh cells (39, 158)) over STAT1 (39), and (iii) an increased expression of the IL-6/IL-6R axis found in chronic SIV (39) provides a molecular basis for the accumulation of Tfh cells in chronic HIV/SIV. Besides Bcl-6, SIV infection induces the expression of c-Maf (157), a master regulator of Tfh cells (139). Interestingly, a higher expression of T-bet (a Th-1 regulator) was found selectively in LN Tfh cells (157), in line with the accumulation of Th1-like Tfh cells in chronic SIV (165). The relative presence of such Tfh populations could have a significant effect on HIV/SIV pathogenesis (165).

Despite the accumulation of Tfh cells (5, 39) and GC B cells (39, 156), the majority of HIV-infected individuals do not develop broadly neutralizing antibodies against HIV (166), pointing to a perturbation of the Tfh-B cell interaction within the GC (167). Increased frequencies of Gag-specific compared to Env-specific Tfh cells found in chronic HIV (5, 39) could reflect a preferential development of Gag-specific Tfh cells or increased turnover of Env-specific Tfh cells. Application of cutting-edge technologies like single cell deep sequencing would be highly informative to this end. Analysis of Simian-Human immunodeficiency virus (SHIV) infected NHPs revealed that besides the frequency, the quality (judged by the expression of IL-21 vs. IFN-γ) of Env-specific Tfh responses was strongly associated with the development of broadly neutralizing antibodies in those animals (168). The increased expression of IL-21 found in HIV-specific Tfh cells (5, 160), could be counterbalanced by a reduced expression of IL-4 by Tfh cells (39), indicating that the development of broadly neutralizing analysis requires the orchestrated expression and activity of relevant cells and soluble mediators.

**Figure 2:**
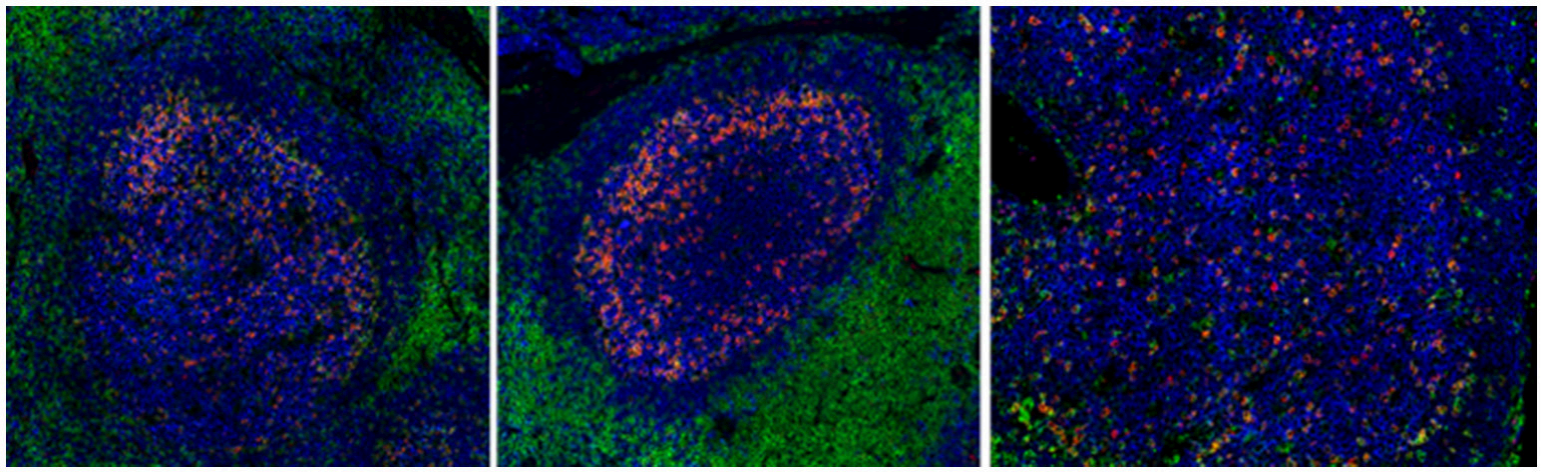
Chronic HIV infection is associated with disturbed follicular organization. Reactive follicles from a healthy axillary (left), a breast cancer SLN (middle) and a HIV viremic axillary LN (right). The CD20 (blue), CD4 (green), and PD-1 (red) markers are shown.

Other mechanisms that could contribute to the impaired functionality of Tfh cells in chronic HIV/SIV include (i) the high expression of PD-L1 on germinal center B cells, interacting with the highly expressed PD-1 on Tfh cells (52), (ii) the relative accumulation of potential suppressor Tfr cells (169) and (iii) the presence of follicular regulatory CD8 T cells (170). Besides the frequency and quality of relevant cells, preservation of the follicular structure is a critical determinant for the development of GC responses in HIV infection- which is characterized by the loss of the Follicular Dendritic Cells (FDC) network and factors secreted by this network, such as CXCl-13 (36). Recent imaging studies revealed that preservation of FDC was associated with maintenance of Tfh cells and preservation of their function in HIV infection, manifested by the response of infected individuals to vaccination (36) and possibly with their distribution within the GC (Figure 2).

HIV/SIV infection affects the dynamics of other LN cells, including CD8 T cells. An increased frequency of follicular CD8 T cells (149, 150, 165) - even within intact follicles (149)- has been observed, implying that infection counteracts local “firewalls” that naturally keep CD8 T cells outside the follicular area. Although the naturally induced HIV-specific cytotoxic CD8 lymphocytes are relatively excluded from the GC area (171), the increased overall presence of follicular CD8 T cells provides an opportunity for novel CD8-based immunotherapies, i.e., the use of bispecific antibodies to redirect these cells to kill HIV-infected cells (149, 172). Immune activation and tissue inflammation are important factors for the dynamics of both follicular CD4 and CD8 T cells in chronic infection (149). The excessive immune activation, however, possibly leads to a generalized, non-cognate driven expansion of these cell populations. One could hypothesize that these dynamics could potentially affect the function of virus-specific Tfh responses, i.e., through the aberrant production of Tfh-cytokines.

## Concluding remarks, future directions

Comparative studies using Tfh cells from diseases with different etiologies represent one way to better understand the molecular and cellular basis for their generation and maintenance. Specifically, such studies between HIV and cancer could inform for:

1. *The mechanisms of Tfh development/maintenance in the settings of a chronic disease*. Besides the relative frequency and spatial positioning, HIV/SIV infection changes the molecular profile of Tfh cells too (39). Is this profile of chronically *in vivo* stimulated Tfh cells unique for HIV/SIV or there is a core, preserved molecular signature during Tfh cell development under different stimuli like cancer neoantigens? Does the etiology/type of cancer have an impact on this profile? Relevant studies will provide critical information about the plasticity of the Tfh cell differentiation program.
2. *The role of tissue inflammation in the development of human Tfh cell responses*. Despite the expected differences between HIV and cancer-including, among others, the (a) nature of antigenic stimulation (viral proteins compared to neoantigens), (b) magnitude/type of tissue architectural changes, (c) presence of virus within the follicle and its ability to infect Tfh cells *per se* in HIV infection- inflammation plays an important role in the pathogenesis of both diseases (172–174). Comparative investigation of LNs from HIV infected individuals and cancer patients could inform for the presence, spatial distribution and possible role of specific pro-inflammatory cellular/molecular mediators for the recruitment of T cell to LNs and their trafficking between LN compartments. General, non-cognate driven activation of LN CD4 T cells could differ between cancer and a chronic viral infection like HIV, with a presumably differential impact in the generation of Tfh cell responses. Therefore, the development of methodologies allowing for the detection of antigen-specific Tfh cells, especially at tissue level, is of great importance.
3. *The impact of tissue structure alterations on Tfh cell dynamics and the and local interplay between adaptive immunity and Tfh cells*. Tissue changes like vasculature reorganization and extracellular matrix organization are less studied in HIV compared to cancer LNs. On the other hand, common tissue structure alterations like fibrosis could contribute to Tfh cell dynamics by affecting the dynamics of innate and adaptive immune cells in the extrafollicular areas (167). HIV/SIV is characterized by the accumulation of LN and particularly follicular CD8 T cells (148, 149). The regulation of these dynamics as well as the role of fCD8 T cells in GC B cell responses is not well understood. Conversely, the role of cytokines, like IL-21 produced by Tfh cells too, as regulators of LN CD8 T cells is not clear either. Understanding the interplay between LN CD8 T cells and Tfh cells is of great importance for LN immune responses both in HIV and cancer, particularly within TLSs, where sequestration of effector CD8 T cells in proximity to the tumor site could have an impact on disease progression.
4. *The “anatomical compartmentalization” of Tfh cell responses*. Are all Tfh cells across the human body the same? Recent studies have shown a differential regulation of Tfh cells between LNs and spleen in SIV infection (156). Is this the case for LN Tfh cells from different anatomical sites too? How that could affect the role of adaptive immunity responses developed in LN in the progression of cancer with different topology?
5. *The assessment of human Tfh cell quality*. Comparison of Tfh cells between HIV donors with broadly neutralizing antibodies and cancer donors with anti-tumor B cell response correlating with good prognosis could help define the spatial organization, the functional characteristics, and molecular signature of an “effective” Tfh cell response.

Given the limited, if any, access to LN tissues, especially from different time points throughout the course of disease, the discovery of circulating biomarkers recapitulating the germinal center reactivity is of great importance. Investigation of molecules like CXCL13 (37, 173) or cTfh cells (113) represents one direction in the hunt for biomarkers i.e., for monitoring the efficacy of vaccination protocols (175). Are cancers of different etiology associated with a particular phenotype/subset of cTfh cells? How do these cells compare to cTfh subsets found in HIV?

An in depth understanding of the Tfh cell development is a prerequisite for the designing of novel *in vivo* interventions aimed at boosting their function and developing effective B cell responses, particularly in HIV. Many questions are still open; how the prime/boost vaccination scheme affects the quality and breath of immunogen-specific Tfh responses? Combining optimal structure-based designed immunogens with new generation adjuvants or interventions targeting specific molecules/pathways involved in the generation of high quality Tfh cells could lead to more efficient vaccine strategies. On the other hand, any therapeutic intervention has to take in consideration the best short and long-term oncological outcome, as well as survivors' quality of life. Surgical interventions may be associated with morbidity/mortality. For example, axillary LN dissection in the context of breast cancer might result in an increased incidence of lifetime lymphedema (up to 21%), decreased arm mobility, and arm paresthesia (174). However, recent clinical trials are challenging the need for axillary surgery for tumors up to 2 cm (175, 176). New therapeutic schemes are urgently needed. The success of cancer immunotherapy in melanoma, lung cancer, renal cell carcinoma and other solid tumors has placed the power of T cell immunity into the armamentarium of cancer therapeutics. Similar immune therapies for patients with breast cancer are beginning to come to fruition, with most promising the use of PD-1 (e.g., pembrolizumab) and PD-L1 (e.g., avelumab) monoclonal antibodies (177, 178). However, the toxicity and cost of immunotherapies has revealed the need for predictive biomarkers. Potential biomarkers should be evaluated in the primary tumor, in the metastases, or in circulation. Given their role in breast cancer, the search for novel biomarkers at a LN and follicular level could be of great support for the decision of relevant surgical procedures.

## Author contributions

AP, YD, TF-H, and ES wrote the manuscript. TC, PL, and CP conceived the idea and participated in the writing and editing the manuscript.

### Conflict of interest statement

The authors declare that the research was conducted in the absence of any commercial or financial relationships that could be construed as a potential conflict of interest.
